# Explosive Weapons Trauma Care Collective (EXTRACCT) Blast Injury Clinical Practice Guideline: Genito‐Urinary Trauma

**DOI:** 10.1002/wjs.12621

**Published:** 2025-05-30

**Authors:** Timothy Craig Hardcastle, Cindy Ann Zietsman, Michael Mwandri, Jana B. A. Macleod, Adama Ouatarra, the EXTRACCT Clinical Practice Guidelines Collaboration

**Affiliations:** ^1^ Clinical Department Trauma and Burns Inkosi Albert Luthuli Central Hospital Durban South Africa; ^2^ Department of Surgical Sciences University of KwaZulu‐Natal Durban South Africa; ^3^ University of KwaZulu‐Natal Durban South Africa; ^4^ Clinical Department of Urology Ngwelezana Hospital KwaZulu‐Natal South Africa; ^5^ ICRC Dar es Salaam Tanzania; ^6^ MSF Dar es Salaam Tanzania; ^7^ Kenyatta University Nairobi Kenya; ^8^ Nazi Boni University Souro Sanou University Hospital Bobo‐Dioulasso Burkina Faso

**Keywords:** assessment, bladder, blast injury, genitourinary, kidney, management, trauma, urogenital

## Abstract

**Introduction:**

This clinical practice guideline from the Explosive Weapons Trauma Care Collective (EXTRACCT) group provides a review of current best practice for the management of urogenital injury after blast injury due to unexploded ordinance and improvized explosive devices. In particular, landmine‐related blasts are a continuing problem and this plagues lower‐income and middle‐income regions disproportionately.

**Methods:**

An expert literature review of current practice is presented.

**Results:**

The guideline provides assessment, resuscitation, and definitive management based on injury severity for the internal urological organs and for the external genitalia. Imaging and surgical techniques are described for the surgeon relatively unfamiliar with the management of urogenital trauma.

**Conclusion:**

Urogenital trauma is seldom fatal but combined with other injuries may cause severe morbidity and mortality. Best practice management is required in resource‐constrained settings.

## 1 Objectives

The objective of this clinical practice guideline (CPG) is to provide the clinician in resource‐challenged civilian environments with an approach to the assessment, diagnosis, and initial management of genitourinary blast‐related injury and provide some guidance on definitive surgical management, including safe transfer to definitive care. It also suggests some aspects of quality assurance and performance improvement strategies for this type of trauma.

## 2 Background

Blast injury from hidden and un‐retrieved landmines, unexploded ordinance, and improvized explosive devices in current and previous conflict zones remains an ongoing challenge to medical care providers in these often resource‐challenged countries, especially for the civilian population. Most civilian clinicians are more comfortable with blunt trauma from motor‐vehicle crashes and interpersonal violence or with penetrating injury from domestic or agricultural implements, whereas intentional violence with knives and low energy gun shots is more common than blast trauma. *Blast injury is a complex cause of trauma, which includes blunt, penetrating trauma, and burns*.

Blast injury is classified as primary (percussive), secondary (penetrating), tertiary (secondary impact), and quaternary (delayed/septic). Recently, a quinary group has been added (systemic effects, such as SIRS/MOFS). Primary genitourinary injury may be in the form of bladder rupture and blunt renal injury. Secondary blast effects may lead to penetrating injury to the soft tissues, including kidney, ureter, and bladder, plus direct genital and perineal injury. Tertiary injury is caused by the body colliding with solid objects when flung by the blast force and again it is the solid organs (kidneys) at risk. Vascular pedicle avulsion or intimal injury may also occur here. Quaternary blast injury to the urogenital tract will mainly be in the form of late sepsis, whereas quinary effects are the consequences of disordered inflammatory responses to the injuries suffered in the prior four phases [1]. In addition to direct genitourinary trauma, the kidneys may suffer from the systemic effects of soft‐tissue injury with acute kidney injury related to crush syndrome, or better termed myonephropathic syndrome, which may confuse the diagnosis due to the “hematuria‐like” pigment nephropathy, resulting in pink to dark brown urine due to the myoglobinuria and subsequent intrinsic renal failure. Genitourinary trauma constitutes about 10% of all injuries and the kidneys or bladder constitute the majority of organs affected. In military epidemiology, perineal injury and kidney trauma were far more common than in civilian practice [2, 3, 4].

Blast injury is no respecter of persons and both adults and children fall victim to the injuries caused by bomb blasts and other explosions. In fact, the risk of thoracoabdominal injury is higher in the age group 6–12 years (average age 10 years), as well as genitourinary injury, whereas mortality is far higher in younger children than adults [5].

### 2.1 Review Method

A literature search with the keywords genitourinary or urogenital, trauma or injury, and blast‐related was undertaken including years 2004–2024 on PubMed on 07/12/2024 that located 104 articles of which on title or abstract review, six articles of relevance to genitourinary trauma were found, and for these, the full‐text was obtained, two additional articles could not be retrieved in full‐text, and were excluded [6, 7, 8, 9]. In addition, one updated European guideline (2023) was found and included [10]. Since trauma to the urogenital system is well described, these new publications were combined with previous work on urological trauma from the Lower‐ and Middle‐Income Country (LMIC) environment from textbooks and publications by the lead author and coworkers [11, 12, 13, 14, 15, 16].

Specific aspects were sought related to blast injury and therefore publications on urological trauma, not mentioning blast injury, may have been missed in the searches, but the content likely to be included was covered in the previous publications. Additionally, the latest version the DSTC manual, published early in 2024, was reviewed for information on urogenital trauma [17].

## 3 Patient Evaluation

Initial assessment and resuscitation [11, 12, 14]:

- Primary survey excluding exsanguinating bleeding with active control of bleeding,
- Management of airway, spinal motion restriction,
- Breathing and ventilation assessment,
- Circulation and other bleeding control,
- Disability assessment and exposure.
- Basic imaging and bloods, which may include ultrasound and plain film Xs‐rays, with the ideal ultimate imaging in blast trauma being a CT‐based trauma “pan‐scan” if this modality is available.
- Secondary survey where the urological and genital tract should be fully evaluated.

More specifically, when the assessment of the urogenital tract is considered, it is important to assess this in the overall clinical picture of the patient. This is paramount to prevent mortality, as genitourinary injury is seldom immediately fatal, with other injuries likely to kill the patient more quickly.

The most common *symptoms and signs* of injury to the genitourinary system are as follows:

- Identify wounds to the external genitalia and perineum and examine and look for flank wounds or bruising,
- Flank pain, anterior lower abdominal pain, fractured lower ribs,
- Macroscopic hematuria, or frank blood at the meatus.
- Microscopic hematuria is important in penetrating trauma, less so with blunt trauma unless hemodynamically abnormal, but may warrant investigation: A positive dipstix should be followed by bedside microscopy to include/exclude cells (hematuria) or not (myoglobinuria).
- Pelvic fracture should prompt investigation due to the high risk for bladder or urethral injury. A cautious attempt at urethral catheterization may be attempted if there is meatal blood, and if failed, imaging is needed to exclude pathology and a suprapubic may be placed under ultrasound guidance considering the possibility of an intraperitoneal rupture in a polytrauma scenario [16].
- Ureteral injuries may be clinically occult, must be excluded in penetrating trauma, while these are highly uncommon in blunt trauma, but more so with secondary and tertiary blast trauma.

Investigations of choice in the unstable patient are

- Semi‐erect chest X‐ray to identify free air or associated lower chest injury which may be associated with renal trauma.
- Ultrasound evaluation for free intra‐abdominal fluid.
- Laboratory tests: renal functions, hematocrit/hemoglobin, and blood crossmatch.
- Urine dipstix in penetrating injury if no macrohematuria is present.
- If time allows, and ultrasound is unavailable, a single‐shot intravenous pyelogram may be done to ensure there is a contralateral functional kidney in case nephrectomy is required, although this has fallen out of favor in recent times [14].
- Placement of a urinary catheter can be diagnostic for hematuria and for resuscitation monitoring.

**FIGURE 1:**
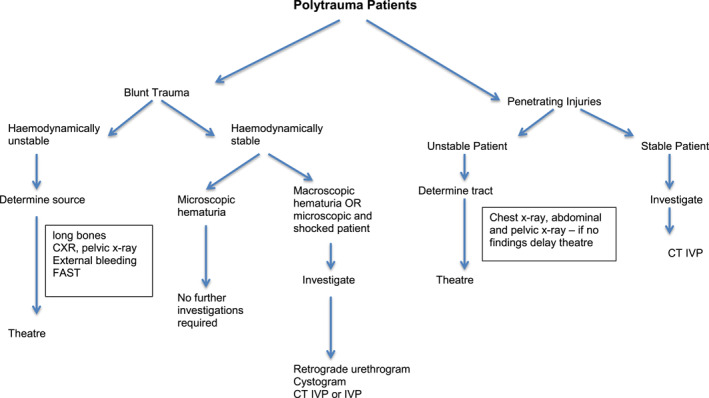
Diagnostic algorithm for urological injury. IVP is the alternative to CT‐IVP, where the latter is unavailable, combined with “FAST” ultrasound screening for free fluid and presence of kidney on the opposite side, including color Doppler for renal flow if available.

For patients responding to resuscitation or hemodynamically normal on arrival: Computerized tomography with intravenous contrast (arterial and portal venous phases) and delayed ureteral phasing, plus a CT cystogram is the investigation of choice. This will demonstrate over 95% of genitourinary tract injuries. Where CT scan is unavailable, the combination of the traditional intravenous pyelography and delayed phase shoot‐through for ureteric integrity may need 2–3 picture captures to be performed consecutively to view all parts of the spindling ureter, together with a formal cystogram and ultrasound of the abdomen to detect free fluid. This is a lower‐cost alternative, as illustrated in Figure 1. A general diagnostic approach is shown in Figure 1. [derived from 14].

## 4 Organ‐Specific Diagnosis and Management for Urological and Perineal Injuries

### 4.1 Renal Trauma [10, 11, 13, 14, 17]

The kidneys are well protected in the retroperitoneum and are more likely injured through direct penetrating injury and less commonly with blunt force application, although more so if lower rib fractures are present or in children where the kidney is more mobile in Gerota's fascia. An actively bleeding renal injury and those with higher grade injury may present unstable and may only be found at exploratory laparotomy. When operation is required for instability, the presence of a renal injury portends to nephrectomy in over 60% of cases [6]. In modern civilian management of renal trauma, the vast majority of kidney injuries are Grades 1–3 and are managed conservatively (See Table 1). Even in the case of some grade IV injuries, conservative management (i.e., avoiding nephrectomy) can be followed, provided high clinical observation is maintained, and when there is evidence for ongoing bleeding or urinary leak, intervention is performed (with either surgery or endovascular options). This approach is also followed for the majority of penetrating injuries as well.

**TABLE 1:** Description of the AAST renal injury grading [16].

Grade	Description of renal injury
I	Contusion or nonexpanding subcapsular hematoma comprises 80% of all renal injuries. No laceration.
II	Non‐expanding perirenal hematoma.
	Laceration < 1 cm deep without urinary extravasation.
III	Cortical laceration > 1 cm without urinary extravasation.
IV	Laceration: Through corticomedullary junction into the collecting system.
	Vascular: Segmental renal artery or vein injury with contained hematoma.
V	Laceration: Shattered kidney.
	Vascular: Renal pedicle injury or avulsion.

If the injury is contained within Gerota's fascia, the tamponade effect and conservative management may include the placement of a JJ stent with a urinary catheter; securing a low‐pressure drainage can lead to a full renal reconstitution after as little as 6 weeks.

**FIGURE 2:**
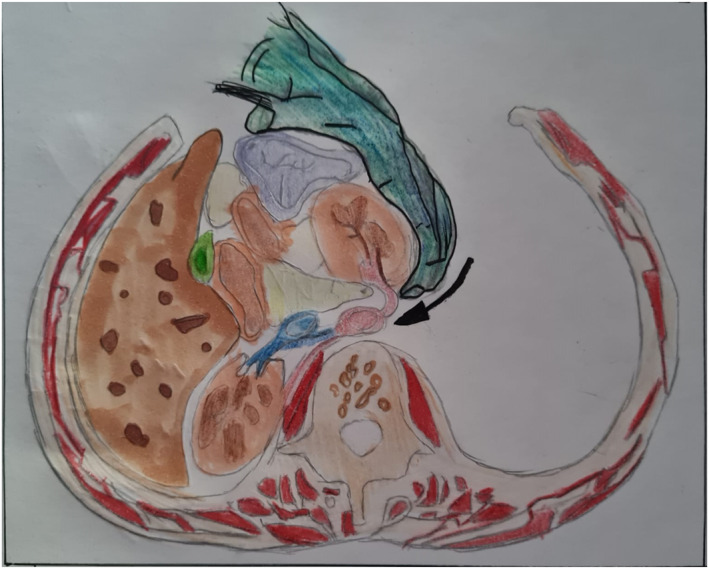
Lateral approach to kidney during nephrectomy for trauma: Spleen, colon, and kidney mobilized to the midline from lateral. (illustration by Jennanice Kershaw).

Operative management is commenced by visualizing the kidneys in Gerota's fascia (without opening the fascia); after performing a right or left visceral rotation as required, and if no expanding or pulsatile hematoma is present, with no visible urine leakage, then the kidney may be managed conservatively (this applies in the civilian sector to both penetrating and blunt injury). If there is a pedicle injury or active bleeding, a possible urinoma noted by expanding hematoma or visible urine leakage, around or engulfing the kidney inside Gerota's fascia, then the Gerota's fascia is opened and the kidney lifted out of the retroperitoneum from lateral to medial, with placement of noncrushing vascular clamps across the hilum (see Figure 2). This is the preferred trauma technique, especially if the patient is unstable, over the traditional approach of first dissecting out the vessels and then opening Gerota's fascia [17]. The latter technique may be considered where there are no other intra‐abdominal injuries, the patient is stable, and there is only isolated renal trauma requiring exploration. In this scenario, the operative repair of the kidney has a much higher success rate. Access to the vascular pedicle may also be effected through the omental bursa.

Most often, renal repair is not possible in the unstable patient and nephrectomy is the procedure of choice, provided the presence of the contralateral kidney is confirmed either by palpation or previously visualized on ultrasound (ideally with Doppler flow checks) preoperatively as the normal size for age. The nephrectomy is performed with ligation of the artery, vein, *and ureter* to prevent urine reflux. If other injuries or physiological instabilities necessitate damage control surgery, temporary abdominal closure with either negative pressure wound closure or a Bogota bag is advised.

For the patient with no other injury indicating the need for surgery and a kidney injury identified on imaging, the preferred approach is nonoperative observation following the guidelines in Box 1.

**BOX 1 Nonoperative management of kidney injury.**

1

- Initial bed rest *until macroscopic hematuria clears* followed by mobilization with minimal strenuous activity for 3 weeks.
- Hydration, to ensure adequate urine output (at least 0.5 mL/kg/hr).
- Monitoring of hematuria, vital signs, abdominal symptoms and signs, and hemoglobin every 12 h for 3–5 days, depending on the clinical condition. Catheterization for 10–14 days with a JJ stent is advantageous.
- Repeat imaging after 2–4 days is only recommended for severe injury (Grades 4 or 5) and/or development of complications.

### 4.2 Ureter Trauma [2, 3, 7, 10, 11, 14, 16, 17]

The ureters are very well protected in the retroperitoneum by the psoas muscle and are seldom injured. Two mechanisms are described—pelvi‐ureteric junction after blunt trauma, which is exceedingly rare, and the more common direct penetrating injury, which is possible anywhere along the course of the ureter. Symptoms are usually minimal and signs may include either micro‐ or macroscopic hematuria, but there may be no clinical markers of injury and thus a low threshold for ureteric imaging is advised, which may be either a “delayed phase” ureteric study at pan‐scan CT or as a ureteric phase IVP in lesser resourced environments. The presence of free fluid on a CT scan or an elevated serum creatinine can be a later‐stage sign of ureteric injury and urine leakage.

If trauma laparotomy identifies a colonic injury or duodenal injury, the ureters must be evaluated by surgical visualization intraoperatively, if not preoperatively imaged. For the injury classification, see Table 2.

**TABLE 2:** AAST ureter injury grading [17].

Grade	Type of injury	Description
I	Hematoma	Contusion or hematoma without devascularization
II	Laceration	Less than 50% laceration
III	Laceration	More than 50% or transection without devascularization
IV	Laceration	Complete transection and less than 2 cm devascularization
V	Laceration	Avulsion with more than 2 cm devascularization

**FIGURE 3:**
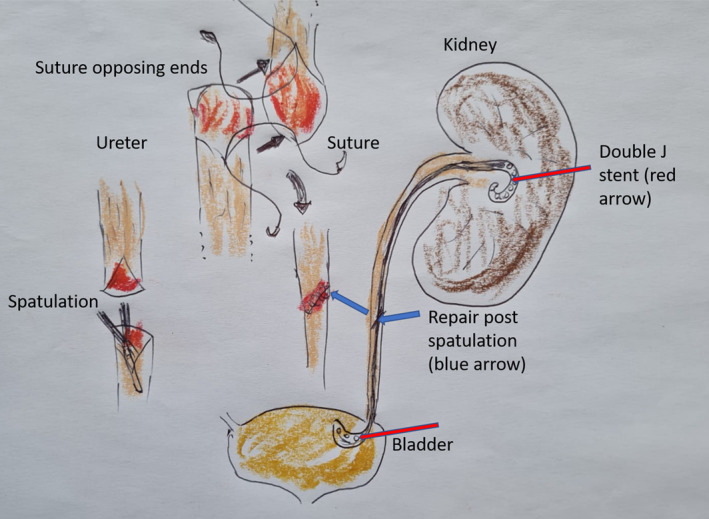
Spatulation repair of the ureter over a double J stent.

Treatment is usually early surgical repair using a spatulation of the ureteric ends to prevent stricturing during healing (see Figure 3), with absorbable sutures preferred (e.g., polyglycolic acid 3/0) and placement of a JJ stent. Treatments differ in the three defined regions of the ureter: proximal 1/3 obtains its blood supply from the medial aspect and therefore preservation of medial tissue and spatulation in accordance with this principle is applied. The mid ureter has the most precarious blood supply and obtains supply from proximal and distal 1/3 and the distal ureter is vascularized through mainly lateral supply and preservation of this must be considered. If doubt exists, then the use of a Boari flap is advocated for the lowest risk of breakdown, or omental wrap buttressing will provide additional blood supply. The JJ stent is left in situ for about 4–6 weeks, and at 3 weeks post repair, a repeat imaging can confirm the absence of a residual leak. If no leak, then the JJ stent is removed in the 4th week, whereas a minor residual leak allows for 3 more weeks and re‐imaging again. If JJ stents are not available, a nephrostomy tube or catheter can be placed with delayed reconstruction. Nonsuction drains in proximity to the repair are advocated in most urology literature, although closed suction systems such as the Blake drain are also acceptable.

More extensive injury may require expert intervention for ureteroureterostomy to the opposite side or reimplantation in the bladder. In these cases, or where damage control surgery is required, the ureter can be exteriorized to control urine leakage.

### 4.3 Bladder Trauma [6, 7, 9, 10, 11, 12, 16, 17]

The bladder is the second most commonly injured genitourinary organ due to its size and location. With landmine explosions and related pelvic trauma, the full bladder can rupture, either extraperitoneal or intraperitoneal or combined. Intraperitoneal rupture always requires surgical repair, whereas the extraperitoneal may on select indication require repair (bone shards in the bladder or prostatic urethral damage), with most cases managed nonoperatively with Foley catheter drainage for 10–14 days.

**FIGURE 4:**
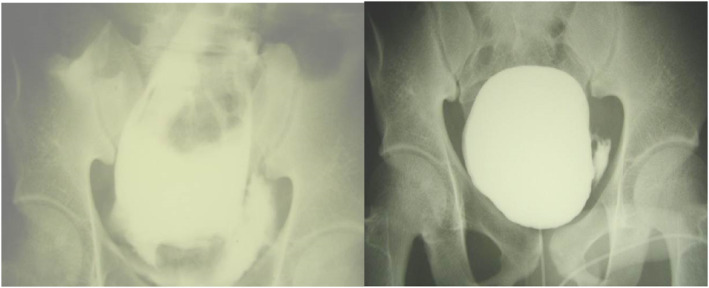
Bladder injuries—intraperitoneal (left) and extraperitoneal (right) on cystogram.

Diagnosis of bladder injury is best confirmed by a formal cystogram using 200–300 mL contrast injected transurethrally into the bladder and then keeping the catheter clamped to prevent the bladder from emptying (See Figure 4). A dynamic view is useful, but a simple pelvic AP and lateral X‐ray with the bladder full will confirm any leaks (contrast extravasation). A post‐micturition set of X‐rays is also advised to see any occult retroperitoneal leaks. Injuries are classified as shown in Table 3.

**TABLE 3:** AAST bladder injury grading [17].

Grade	Type	Description
I	Hematoma Laceration	Contusion/intramural hematoma Partial thickness laceration
II	Laceration	Extraperitoneal less than 2 cm
III	Laceration	Extraperitoneal > 2 cm or intraperitoneal < 2 cm
IV	Laceration	Intraperitoneal > 2 cm laceration
V	Laceration	Any of the above involving the bladder neck or urethral trigone

Repair is either at midline laparotomy or via a Pfannestiel approach (for isolated bladder injury) opening the bladder dome (if not clearly ruptured) and intra‐cystic repair of any deep injury followed by the primary closure of the dome. At all times, the ureteric and urethral orifices must be visualized during bladder repairs, with stents placed or a small feeding tube inserted to provide visualization. During laparotomy or when the injury is near the dome, the bladder can be repaired directly without opening in two layers. A rapidly absorbable suture (e.g., coated polyglycolic acid) is preferred and a *trans*‐urethral catheter is left for 10–14 days to allow healing. A repeat cystogram at day 10 post repair may be considered, but is not mandatory. Retropubic closed suction (e.g., Blake) or nonsuction drains are advocated, removed when no longer draining and healing confirmed on repeat imaging.

### 4.4 Urethral Injury [10, 11, 15, 16, 17]

Urethral injury has traditionally been associated with blunt straddle injury and pelvic fractures. In blast trauma, the effect of the blast on the lower limbs and perineum is more extensive, injuring the scrotum, vulva, and avulsing the surrounding tissues to the extent of penetrating or devitalizing the entire area, often exposing or tearing the actual urethra, mainly in the male; but with extensive perineal blast injury, even the short female urethra may be injured. These extensive injuries are much less common in civilian practice and are classified as depicted in Table 4.

**TABLE 4:** AAST urethral injury grading [17].

Grade	Type	Description
I	Contusion	Blood at meatus, with normal RUG
II	Stretch	Elongation of urethra without RUG contrast extravasation
II	Partial disruption	Extravasation of contrast at the injury with continuity to the bladder
IV	Complete disruption	Extravasation of contrast at the injury without bladder contrast and less than 2 cm separation
V	Complete disruption	Complete disruption at the injury without bladder contrast and more than 2 cm separation or involving prostate or vagina

**FIGURE 5:**
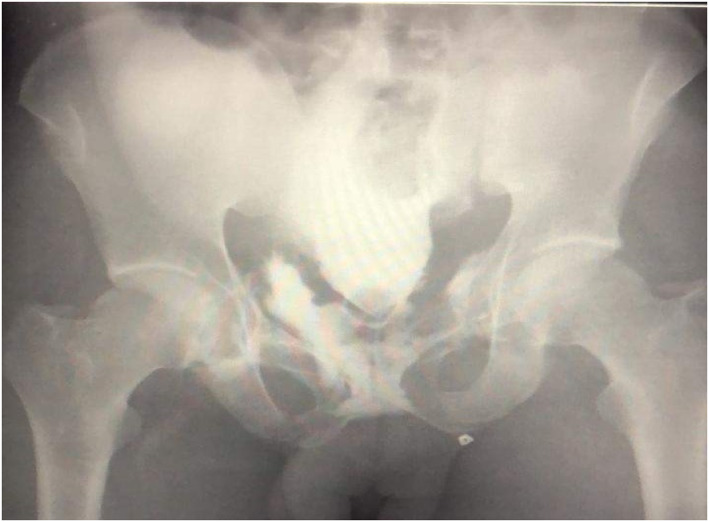
Retrograde urethrogram (RUG) showing urethral extravasation and partial urethral injury.

**FIGURE 6:**
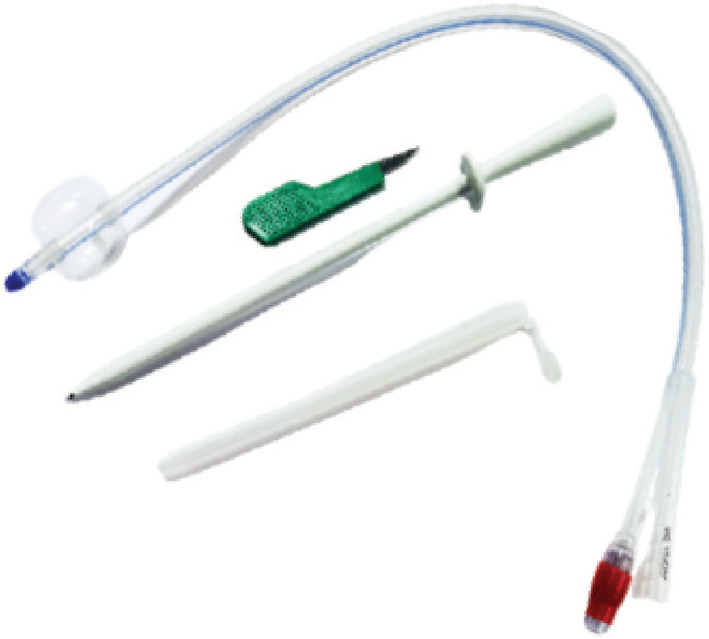
Suprapubic catheter placement set (one example): trocar, bistoury, sheath, and catheter.

Blast can also cause pelvic fractures increasing the risk of urethral injury. Blood at the meatus and inability to pass a transurethral catheter are the mainstays of diagnosis. Retrograde urethrography (RUG) is performed, in stable patients only, by placing a small catheter into the fossa navicularis of the glans penis and injecting contrast under dynamic imaging (fluoroscopy)—see Figure 5. If not available, a single‐shot bedside RUG can be performed. Any contrast extravasation should prompt suprapublic catheter placement (see Figure 6). If there is an experienced person available and the patient is stable, then a single attempt at gentle transurethral catheter placement may be made, using a larger rather than a smaller catheter, to serve as a stent and prevent catheter curling in the distal urethra.

The optimal treatment is early realignment surgery using urethroscopic placement of a transurethral catheter, but this requires expertise and equipment. Open surgical realignment (via the bladder) at a trauma laparotomy using magnetic “sounds” to connect the ends of the rupture and then railroad a transurethral catheter over a guidewire is another option. Leaving an additional suprapubic catheter is a safe temporary option during the healing process.

### 4.5 Penile and Scrotal Injury [4, 6, 8, 11, 15, 16, 17]

**FIGURE 7:**
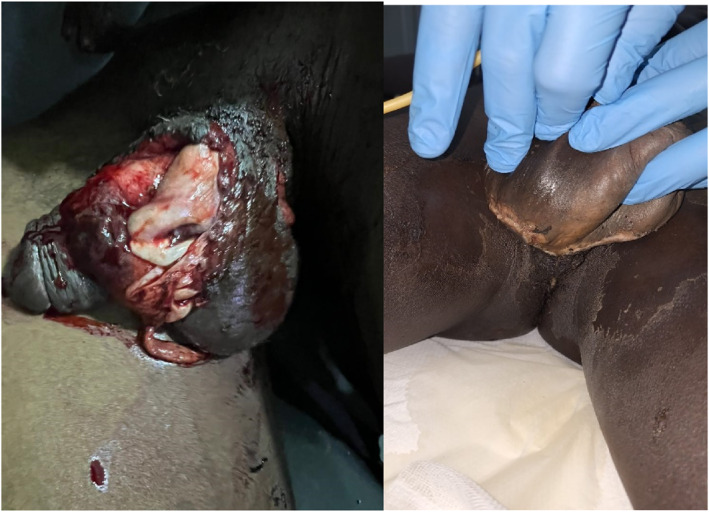
Soft tissue damage from a high energy gunshot, with delayed presentation: Debridement and tissue closure is key to management.

The penis consists of the extra‐perineal urethra ending on the glans penis, surrounded by the corpus spongiosum and two corpora cavernosae with the arterial plus venous and nerve supply all covered by Buck's fascia that is contiguous with the dartos fascia. The scrotum consists of skin covering the two testes, which are encapsulated in their tunica vaginalis and dartos muscles, with the epididymis and vas deferens in the surrounding soft tissue. Blast injury to the penis can avulse the tissue layers causing vascular injury to the testis, avulsion of the testis, urethral penetration, and corporal bleeding. Skin loss may leave the testis exposed and this is addressed by implantation in viable soft tissue, if available on the thigh or the abdominal wall. See Table 5 for the injury severity descriptions. Regular debridement, washouts with suitable antiseptic solutions, dressing changes, and the use of negative pressure wound care are advised to allow for coverage and delayed healing. Appropriate antibiotic therapy is recommended for contaminated wounds or if associated with open fractures (see Figure 7).

**TABLE 5:** AAST penile and yestis injury grading.

Grade	Testis description	Penis description
I	Contusion	Cutaneous laceration
II	Subclinical laceration of tunica	Corpus cavernosum laceration without tissue loss
III	Tunica laceration with < 50% tissue loss	Cutaneous avulsion, or laceration through glans or cavernosal defect < 2 cm
IV	Major laceration with > 50% tissue loss	Partial penile avulsion
V	Testicular destruction or avulsion	Total penile avulsion

Given the generally good blood supply, using scrotal Doppler ultrasound is advised to check testicular perfusion and liberal debridement, and the closure of wounds is the optimal treatment. Reconstruction in layers is advised where the tissue is viable and sufficient to cover the defect, usually done over a transurethral catheter. For testes injury, Grade III or higher debridement and tunica closure (when possible) may be performed, or orchiectomy, when the testis is clinically nonsalvageable. Where all the tissues are avulsed, a suprapubic catheter is placed and the wound allowed to heal by delayed intention. The proximal urethra may be diverted to the surface if there is sufficient skin and attached to the skin to allow for seated voiding as a perineal urethrostomy until the tissue has healed and secondary reconstruction may be considered—this technique is often used in urethral stricture staged repairs or hypospadias repairs. Perianal skin should be loosely approximated to counter the natural contractures of the tissue that may interfere with delayed repair. If the wound also involves the perianal tissues, a diverting loop colostomy is advised. The corpora cavernosae should be repaired in a watertight fashion using absorbable sutures to avoid the complications of penile deformity and inability to achieve an erection. Avulsed penile tissue may require reimplantation by experts and referral is advised.

Testicular viability is best assessed with color Doppler ultrasound, and any vascular impairment should be addressed early.

### 4.6 Female Injury Specifics [2, 3, 4, 15, 17]

**FIGURE 8:**
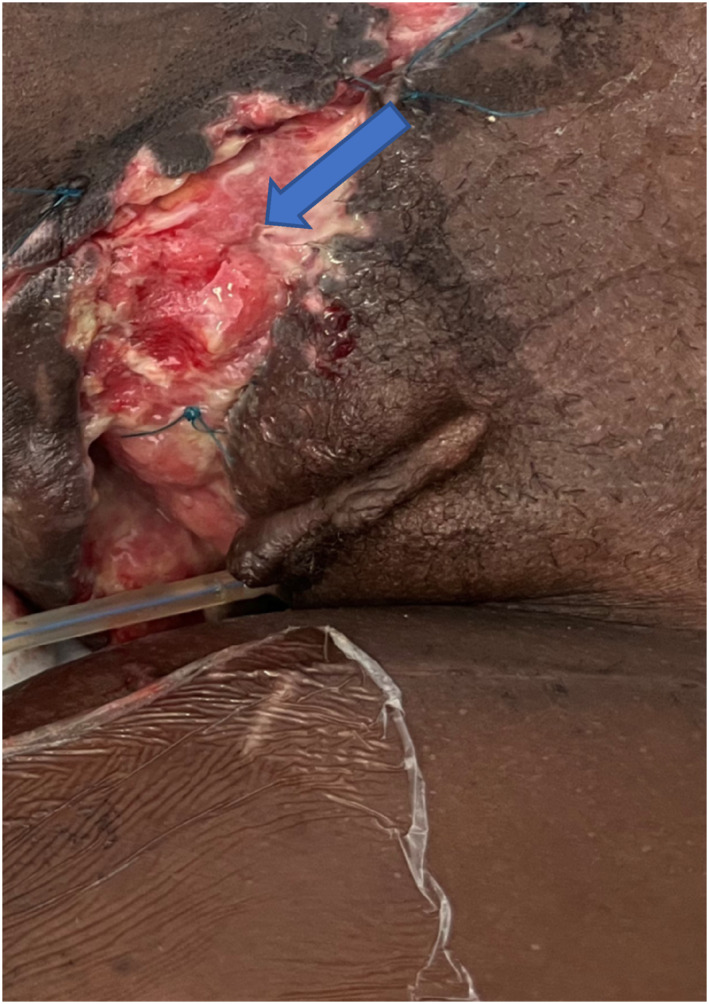
Female blunt perineal and genital injury—exposed labia majorum (arrow).

**FIGURE 9:**
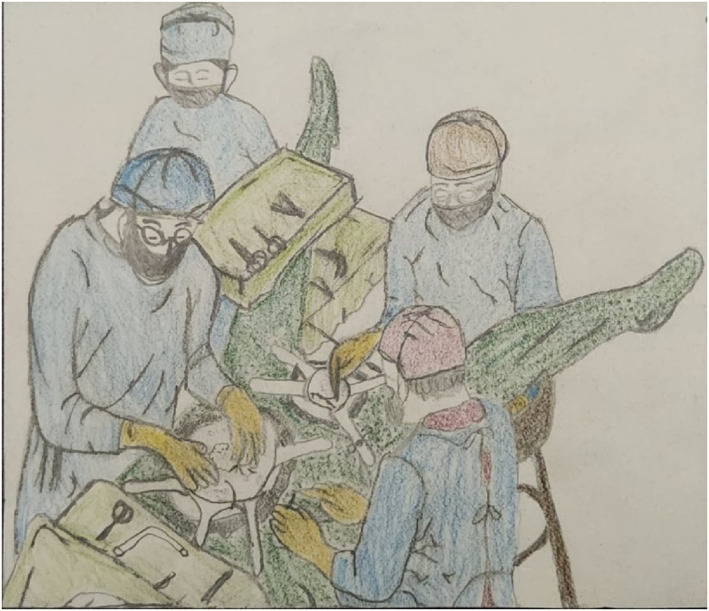
Illustration of Lloyd‐Davies position for perineal access (illustration by Jonathan Hardcastle).

The female perineum is much less exposed; however, with landmine blast and IED‐related injury, the risk exists for the involvement of the female perineum in destructive injuries (see Figure 8 and Table 6). Hemorrhage control is paramount. The vagina, vulva, and their close proximity to the bladder and rectum necessitate an extensive examination to exclude perforating injuries, best performed in the Lloyd‐Davies (partial lithotomy) or lithotomy position (see Figure 9). The vulva should be debrided, the female urethra catheterized, and the perianal musculature evaluated. Urethral avulsion from the bladder neck has been reported and this is repaired over a transurethral catheter. Any risk of combined genito‐rectal injury should lead to diverting loop colostomy and, if needed, suprapubic catheter placement to divert fecal and urine from the tissues to allow healing. Careful examination of any patient presenting either early or late with incontinence needs to exclude a rectovesical or rectoureteric fistula and in women a vesicovaginal, ureterovaginal, or ureterouterine, or even a vesicouterine fistula must be excluded and managed usually by diversion followed by delayed (6 months minimum timeframe) repair and reconstruction.

**TABLE 6:** Female external genital injury scale.

Grade	Vulva	Vagina
I	Contusion or hematoma	Contusion or hematoma
II	Superficial skin laceration only	Mucosa only laceration
III	Deep laceration into fat or muscle	Deep laceration into fat or muscle
IV	Avulsion of skin, fat or muscle	Laceration to cervix or into peritoneum
V	Extending into adjacent organs: anus, rectum, urethra, or bladder	Extending into adjacent organs: anus, rectum, urethra, or bladder

## 5 Considerations in Transport and Safe Transfer [10, 11, 15, 16, 17]

The patient who is conscious and has multiple injuries should be offered appropriate immobilization, pressure dressings on bleeding sources, once external bleeding has slowed by direct pressure, and prior to transfer, ensure that airway, breathing and hemodynamics have been optimized to ensure safe arrival at the eventual destination. Catheters should be filled with water or saline and not air, so as to prevent changing catheter bulb dimensions during air transfer. Safe drainage of urine, stool, and prevention of wound contamination should be optimized. All documentation and a summary of the vital signs, blood investigations performed and their results, interventions and treatment undertaken, and imaging results must accompany the patient to a higher level of care. Monitoring urine output is essential. Appropriate analgesia, with preferably ketamine or opioids, should be provided, with ketamine producing hemodynamical variability.

### 5.1 Performance Improvement

Regular audit of practice, assessment of times to resuscitation, diagnosis, and intervention with feedback to clinicians and the community will enhance the outcome of these treatments. Practical implementation of audit findings can improve the systems of care.

## 6 Conclusion

This CPG has provided an overview of the approach to the assessment, diagnosis and management options for injuries from blast‐related trauma to the entire genitourinary system and related external structures.

## Author Contributions

**Timothy Craig Hardcastle:** conceptualization, methodology, supervision, writing – original draft, writing – review and editing, project administration. **Cindy Ann Zietsman:** investigation, formal analysis, writing – review and editing, data curation. **Michael Mwandri:** investigation, validation, data curation, writing – review and editing. **Jana B. A. Macleod:** writing – review and editing, formal analysis, data curation, methodology. **Adama Ouatarra:** visualization, validation, writing – review and editing, data curation.

## Ethics Statement

The authors have nothing to report.

## Conflicts of Interest

There are no financial or other conflict of interest for any of the authors, except that T.C.H. is an associate editor of the *World Journal of Surgery*.

## Data Availability

The data that support the findings of this study are available on request from the corresponding author. The data are not publicly available due to privacy or ethical restrictions.
